# Clinicopathological and Immunomicroenvironment Characteristics of Epstein–Barr Virus-Associated Gastric Cancer in a Chinese Population

**DOI:** 10.3389/fonc.2020.586752

**Published:** 2021-01-08

**Authors:** Xiaoxia Jia, Ting Guo, Zhemin Li, Meng Zhang, Yi Feng, Bin Dong, Zhongwu Li, Ying Hu, Ziyu Li, Xiaofang Xing, Shuqin Jia, Jiafu Ji

**Affiliations:** ^1^Department of Molecular Diagnosis, Key Laboratory of Carcinogenesis and Translational Research (Ministry of Education), Peking University Cancer Hospital & Institute, Beijing, China; ^2^Department of Gastrointestinal Translational Research, Key Laboratory of Carcinogenesis and Translational Research (Ministry of Education), Peking University Cancer Hospital & Institute, Beijing, China; ^3^Department of Gastrointestinal Surgery, Key Laboratory of Carcinogenesis and Translational Research (Ministry of Education), Peking University Cancer Hospital & Institute, Beijing, China; ^4^Department of Pathology, Key Laboratory of Carcinogenesis and Translational Research (Ministry of Education), Peking University Cancer Hospital & Institute, Beijing, China; ^5^Biobank, Key Laboratory of Carcinogenesis and Translational Research (Ministry of Education), Peking University Cancer Hospital & Institute, Beijing, China

**Keywords:** Epstein–Barr virus (EBV), gastric cancer, CD3, CD68, immune microenvironment

## Abstract

**Background:**

Epstein–Barr virus-associated gastric cancer(EBVaGC)has a unique tumor immune microenvironment. We performed a comprehensive analysis of the tumor-infiltrating immune cells in a cohort of EBVaGC in a Chinese population.

**Methods:**

Epstein–Barr encoding region (EBER) *in situ* hybridization was performed in 1,328 consecutive cases of surgically resected GC. Densities of immune cells, including T cells, B cells, natural killer cells, and macrophages from the patients were calculated after immunohistochemical staining with CD3, CD20, CD57, and CD68 antibodies in tissue microarrays, respectively.

**Results:**

EBVaGC patients accounted for 4.1% (55 of 1,328) cases in the overall population. The average age of patients with EBVaGC was lower than that of non-EBVaGC patients. Histologically, EBVaGC patients exhibited poorly differentiated adenocarcinoma (P = 0.004) and lower frequency of vascular invasion (P = 0.034). The density of CD3^+^ T lymphocytes (CD3, 23.84 ± 14.49 *vs.* 12.76 ± 8.93, P < 0.001) and CD68^+^ macrophages (CD68, 9.73 ± 5.25 *vs.* 5.44 ± 4.18, P < 0.001) was significantly higher in EBVaGC patients. CD3^+^ T cell density predicted better 5-year overall survival of EBVaGC patients (P = 0.022).

**Conclusions:**

EBVaGC patients were younger with low-differentiated adenocarcinoma and less vascular invasion. Increased infiltration of multiple immune cells affected the prognosis of patients, especially EBVaGC patients with more CD3^+^ T lymphocytes, who survived longer.

## Introduction

Approximately 4–18% of the cases of gastric cancer (GC) are caused by Epstein–Barr virus (EBV) (1). EBV infection occurs at the initial or early stage of carcinoma development. The Cancer Genome Atlas of 2014 divided GC into the following four types according to characterization based on molecular biology: EBV-associated gastric cancer (EBVaGC), microsatellite unstable tumors, genomically stable tumors, and chromosomally unstable tumors. The molecular characteristics of EBV-positive gastric cancer were summarized and attributed to mutation of phosphatidylinositol-4,5-bisphosphate 3-kinase catalytic subunit alpha (PIK3CA), overexpression of programmed death-ligand 1 and 2 (PD-L1/2), EBV-CIMP (CpG island methylator phenotype), silencing of cyclin dependent kinase inhibitor 2A (CDKN2A) (p16^INK4A^), and immune cell signaling (2). The clinical benefit of EBVaGC patients after anti-PD-L1 antibody treatment can be maintained for a longer period of time. In patients with EBV-positive tumors, dramatic responses to pembrolizumab were observed (objective response rate of 100% in EBVaGC), which might be related to EBVaGC-rich immune cell infiltration and increased expression of immune checkpoint pathway genes (3–5). Therefore, EBV-positive status may be a potential biomarker for GC immunotherapy. Analysis of the immune microenvironment of EBVaGC is necessary.

The effect of EBV infection on GC cells is complicated by various immune cells in the tumor immune environment. EBVaGC cells express high levels of PD-L1 and inhibit T cell proliferation with the involvement of the interferon-gamma pathway (6). Increased expression of C-C motif chemokine 22 in EBVaGC can attract more regulatory T cells (Tregs) (7). Expression levels of genes involved in antigen presentation are also significantly upregulated in EBVaGC. The authors also described the significant increase in the expression of co-stimulatory molecules involved in T cell activation and survival in EBVaGC (8). Compared with non-EBVaGC, EBVaGC patients reportedly exhibit increased mRNA expression profiles of immune-related genes, including increased expression of cytotoxic T cells, Th1 cells and pro-inflammatory factors, as well as Tregs and immune suppression checkpoint gene expression (9).

EBV infection can trigger a significantly higher infiltration of CD8^+^ T cells and cytotoxic T lymphocytes (CTLs) in EBVaGC (10). The number of infiltrating CD8^+^ T cells in EBVaGC always exceeds the number of infiltrating CD4^+^ T cells, usually reaching a ratio of 10:1 (11). Our previous study suggested that the survival of EBVaGC patients could be significantly extended by the high expression of PDL1 (12). Another study reported that EBV tumors were most infiltrated with CD8^+^ CTL and macrophages (28 and 22% of all intratumoral cells, respectively), with an intermediate frequency of CD4^+^ T cells (20%) and low rate of Tregs (4%) (13). A high density of CD204^+^ tumor-associated macrophages has been associated with the aggressive GC tumor behavior and worse survival of GC patients. Low density of CD204^+^ tumor-associated macrophages is associated with EBV infection, which may explain the favorable outcome of EBV-associated gastric carcinoma (7). The number of dendritic cells in EBVaGC is also higher than that in non-EBVaGC (9). Tumor-associated stromal cells are involved in the formation of the EBVaGC immune environment (14). The density of neutrophils in the EBVaGC tumor microenvironment is markedly low. In some cases, neutrophils are not detected in the tumor tissue.

However, most studies on the EBVaGC tumor microenvironment have focused on certain cytokines or immune cells. A comprehensive analysis and overall research on individual cells and components in the immune environment are unavailable. Therefore, we analyzed immune cells, including T cells, B cells, natural killer (NK) cells, and macrophages in the tumor microenvironment. These analyses were combined with the analysis of the clinical features of EBVaGC from 1,328 consecutive cases of surgically resected GC. The findings reveal more about the immune characteristics of GC and suggest better treatments to improve survival.

## Materials and Methods

### Study Population

Patients who underwent curative gastrectomy for adenocarcinoma of the stomach or esophageal-gastric junction at Peking University Cancer Hospital between January 2008 and December 2012 were eligible. The three criteria for inclusion in this study included availability of formalin-fixed, paraffin-embedded tissues, histologic identification of adenocarcinoma, and no history of preoperative chemotherapy or radiotherapy. This study was approved by the institutional review board of Peking University Cancer Hospital. Written informed consent was obtained from all patients. The pathological tumor-node-metastasis (pTNM) stage was determined according to the 7th edition of the Union for International Cancer Control guidelines.

### Tissue Microarray

The tissue microarray used in this study included 1,328 unselected, primary, sporadic GCs. According to the histopathological classification system adopted by the World Health Organization, all hematoxylin and eosin (H&E)-stained slides were examined in the pathology department of Peking University Cancer Hospital to confirm the tumor type and degree of differentiation. A representative area of each tissue sample was selected and carefully marked on the H&E-stained sections. Three representative core tissue samples (1 mm diameter) were punched out from the corresponding single donor tissue block and rearranged in the recipient block. Each TMA spot contained at least 50% tumor cells (15).

### Immunohistochemistry and Evaluation

Continuous 4 μm-thick tissue array sections were obtained and mounted on glass slides. Before the high-throughput immunohistochemistry procedure, the slides were baked at 60°C for 2 h. The arrays were dewaxed by washing sequentially with xylene, gradient ethanol, and water. Antigens were recovered at 95°C for 15 min. Endogenous peroxidase was blocked with 3% hydrogen peroxide for 30 min. Block non-specific staining was performed using 10% normal goat serum (in 1× PBS) at 37°C for 1 h Following antigen retrieval, the samples were incubated overnight at 4°C with the following primary antibodies: anti-CD3 (NCL-CD3-565, Leica, Wetzlar, Germany), anti-CD20 (L26, DAKO), anti-CD68 (Z2071, Zeta Corporation, Sierra Madre, CA, USA), anti-CD57 (TB01, DAKO), anti-PD-L1 (SP142, all purchased from Roche, Basel, Switzerland), anti-proliferating cell nuclear antigen (PCNA) (CBL407, Millipore, Darmstadt, Germany), and anti-HER2 (clone 4B5, Roche). Normal IgG was used as the negative control. The ABC kit (DAKO, Glostrup, Denmark) was used to perform the enhancement step as per the manufacturer’s instructions. Samples were then incubated with the secondary antibody (1 h at room temperature) and the diaminobenzidine substrate (5 min at room temperature). The samples were subsequently dehydrated in a gradient series of ethanol and counterstained with hematoxylin for 2 min at room temperature. The slides were then rinsed, cleared, and mounted. The staining of each antibody was optimized based on negative and positive controls (15).

Human epidermal growth factor receptor 2 (HER2) immunoreactivity was scored following the HER2 scoring scheme (scores of 0, 1^+^, 2^+^, and 3^+^) according to HER2 overexpression assessment for GC. To evaluate PCNA expression in tumor cells, the average values were stratified into the following three scoring groups: 1, not detected or <10% positive cells; 2, 10–20% positive cells or 20–50% weakly positive cells; and 3, 20–50% positive cells with moderate to marked reactivity or >50% positive cells. An immunoreactivity scoring system consisting of two categories was applied for the evaluation of PD-L1 expression in tumor cells. Category A was used to rate the percentage of immunoreactive cells and was graded as 0 (negative, or <1 positive), 1 (1–10% positive), 2 (10–50%), and 3 (>50%). Category B was used to document the intensity of immunostaining as 0 (no immunostaining), 1 (weak), 2 (moderate), or 3 (strong) (12).

### Epstein–Barr Virus -Encoded Small RNA In Situ Hybridization

All the tissue slides were subjected to ISH using the BIO-HRP REMBRANDT® EBER RISH kit (PanPath B.V., Budel, Netherlands) according to the manufacturer’s instructions. A known EBV-positive Burkitt’s lymphoma was used as the positive control. Hybridization without a probe was performed and considered as the negative control.

### Evaluation of CD3, CD20, CD68, and CD57

Stained slides were scanned at ×20 magnification using the Aperio XT digital slide scanner (Leica) and subjected to automated image analysis to detect and quantify immunoreactivity. An in-house developed software system, designated TMAi, was used to distinguish between brown (immunopositive) pixels, blue (immunonegative) pixels, and white (empty space) pixels. All tissue cores were reviewed after the image analysis process by a senior gastrointestinal histopathologist to confirm that the software detection of the brown DAB staining was accurate and to exclude all cores that contained tumor cells. The percentage immunoreactivity was calculated as positive cells (positive cells + negative cells) × 100. The values from all available cores per case were averaged and used as a surrogate for the extent of immune cell infiltration. The cutoff values to classify CD3, CD68, CD30, and CD57 constituted the third quartile (CD3, NCL-CD3, Leica; CD68, Z2071, Zeta Corporation; CD57, TB01, DAKO; and CD20, L26, DAKO, L26).

### Statistical Analyses

Correlation analysis was assessed by the Fisher’s exact or the Cochran–Mantel–Haenszel 2 test. Overall Survival (OS) was calculated using the Kaplan–Meier method. The log rank test was used to determine significance of differences. Hazard ratios of variables were calculated by univariate Cox regression model. HRs with P-values ≤0.05 were included in a multivariable Cox regression, combined with the iterative backward LR method was used to identify independent prognostic variables. All statistical tests were two-sided at the 5% level of significance. The false discovery rate was controlled by applying the explorative Simes (Benjamini–Hochberg) procedure group-wise for each biomarker. The manuscript was written in accordance with the criteria specified in the reporting recommendations for tumor marker prognostic studies (REMARK). Statistical analyses were performed with SPSS 24.0 (IBM Corporation, Armonk, NY, USA).

## Results

### Clinicopathological Features of Epstein Barr Virus-Associated Gastric Cancer

**Figure 1:**
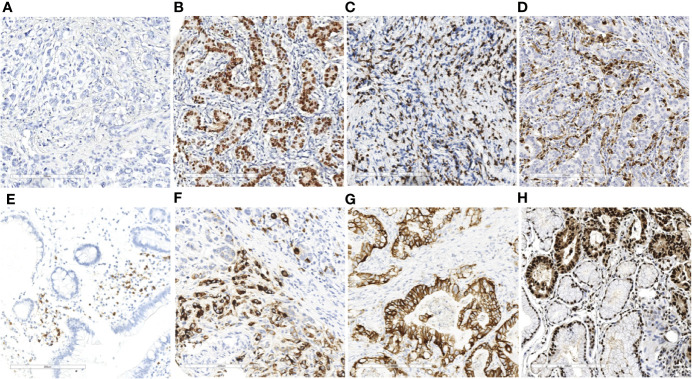
EBER ISH and immunohistochemistry results of immune markers in gastric cancer (×20). EBER ISH-negative **(A)** and EBER ISH-positive **(B)** cases. Representative images of CD3^+^ T lymphocytes **(C)** CD68^+^ macrophage **(D)** CD20^+^ B lymphocyte cells **(E)** and CD57^+^ NK cells **(F)** and expression levels of HER2 **(G)** and PCNA **(H)** in gastric cancer tissues.

The EBER ISH results revealed that 55 of 1,328 GCs (4.1%) were classified in the EBVaGC group (**Figure 1**). EBVaGC was frequently found in patients <60 years of age (5.4 *vs.*2.8 %, P = 0.017, **Table 1**). Poorly differentiated cancer was more frequent in EBVaGC patients (6.3 *vs.* 2.7 *vs.* 0.0%, P = 0.004, **Table 1**) and adenocarcinoma was more frequent than Ring cell carcinoma (4.5 *vs* 0.0%, P = 0.029, **Table 1**). The frequency of vascular invasion was lower in the EBVaGC group than that in the non-EBVaGC group (5.5 *vs.* 3.1%, P = 0.034, **Table 1**). There were no statistically significant differences in the expression of PCNA (P = 0.180) and HER2 (P = 0.736) between EBVaGC and non-EBVaGC patients.

**Table 1:** Clinical and molecular characteristics of patients according to EBER.

Characteristics	EBER		P-value
	Negative	Positive	
**All cases**	1273	55	
**Age**			0.017
<=60	647(94.60%)	37(5.40%)	
>60	626(97.20%)	18(2.80%)	
**Gender**			0.174
Female	336(97.10%)	10(2.90%)	
Male	937(95.40%)	45(4.60%)	
**Lauren type**			0.101
Diffuse	297(95.80%)	13(4.20%)	
Intestinal	720(96.40%)	27(3.60%)	
Mixed	200(93.00%)	15(7.00%)	
**Differentiation**			0.004
Poor	581(93.70%)	38(6.30%)	
Moderate	584(97.30%)	16(2.70%)	
Well-differentiated	31(100.00%)	0(0.00%)	
**Tumor size**			0.966
<5cm	657(95.60%)	30(4.40%)	
>=5cm	554(95.70%)	25(4.30%)	
**Vascular invasion**			0.034
Negative	605(94.50%)	35(5.50%)	
Positive	630(96.90%)	20(3.10%)	
**Histological type**			0.029
Adenocarcinoma	1158(95.50%)	55(4.50%)	
Signet ring cell carcinoma	101(100.00%)	0(0.00%)	
**pTNM**			0.259
0	1(100.00%)	0(0.00%)	
1	139(97.90%)	3(2.10%)	
2	361(94.50%)	21(5.00%)	
3	600(95.40%)	29(4.60%)	
4	109(98.20%)	2(1.80%)	
**PCNA**			0.180
1	40(100.00%)	0(0.00%)	
2	140(97.20%)	4(2.80%)	
3	907(95.00%)	48(5.00%)	

### Association Between CD3, CD20, CD68, CD57 and Clinicopathologic Parameters

**Figure 2:**
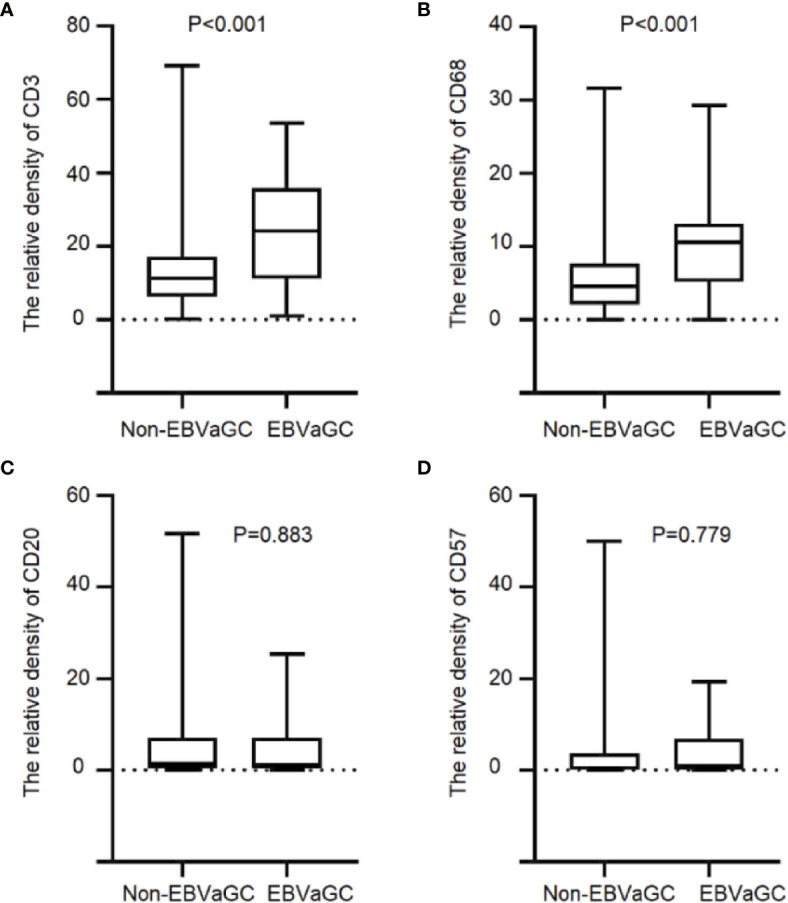
Immune cell infiltration in EBVaGC and non-EBVaGC. The panels show the density of **(A)** CD3^+^T lymphocytes.**(B)** CD68^+^ macrophages, **(C)** CD20^+^ B lymphocytes, and **(D)** CD57^+^ NK cells.

We quantified T lymphocyte density of CD3^+^, B lymphocyte cell density of CD20^+^, macrophage density of CD68^+^ and NK cell density of CD57^+^ to characterize the immune infiltrate in EBVaGC cases (**Table S1**). We performed IHC detection on CD3^+^T cells, CD68^+^ macrophages, CD20^+^ B cells and CD57^+^ NK cells in the tumor tissues of 1,328 patients.CD3^+^T cells and CD68^+^ macrophages infiltration increased significantly in EBVaGC patients (CD3^+^, mean ± SD, 23.84 ± 14.49 *vs.* 12.76 ± 8.93, P < 0.001, **Figure 2A**; CD68^+^, 9.73 ± 5.25 *vs.*5.44 ± 4.18, P < 0.001, **Figure 2B**). No significant differences were evident between EBVaGC and non-EBVaGC patients regarding the density of CD20^+^ B cells (4.59 ± 6.38 *vs.* 4.87 ± 7.16, P = 0.883, **Figure 2C**) and CD57^+^ NK cells (CD20^+^, 3.23 ± 5.05 *vs.* 3.67 ± 6.82, P = 0.779, **Figure 2D**). Compared with patients who did not receive chemotherapy, the density of CD3^+^ T lymphocytes (23.077 ± 14.353 *vs.* 25.75 ± 15.226, P = 0.565) and CD68^+^ macrophages (9.685 ± 4.718 *vs.* 9.852 ± 7.002, P = 0.923) in the tumor tissue of patients receiving neoadjuvant chemotherapy was lower. While the density of CD20^+^ B lymphocytes (5.23 ± 6.989 *vs.* 25.75 ± 15.226, P = 0.314) and CD57^+^ NK cells (4.065 ± 5.57 *vs.* 25.52 ± 3.804, P = 0.315) in the tumor tissue of patients receiving neoadjuvant chemotherapy was higher. Although the differences were not statistically significant, there was an apparent effect of neoadjuvant chemotherapy on the tumor immune microenvironment (**Table S2**).

In the treatment-naïve EBVaGC tissues (**Table 2**), CD57^+^ NK cells density was significantly higher in older patients with EBVaGC (P = 0.032). CD20^+^ B lymphocyte cell infiltration was significantly higher in female patients (P < 0.001). CD68^+^ macrophage density was positively associated with tumor size (P = 0.022), whereas the density of CD57^+^ NK cells was much lower in EBVaGC samples with a tumor diameter greater than 5 cm (P = 0.026). Regarding PD-L1 expression in EBVaGC, infiltration of CD57^+^ NK cells was negatively correlated with evaluation of PD-L1 expression based on staining of tumor cells (PD-L1-TU) (P=0.016). CD3^+^T cell density showed a relatively higher trend (P = 0.094) in PD-L1-TU positive EBVaGC patients.

**Table 2:** Correlation between density of tumor-infiltrating immune cells and clinicopathologic features of EBVaGC patients.

Characteristics	CD3		P-value	CD68		P-value	CD20		P-value	CD57		P-value
	Low	High		Low	High		Low	High		Low	High	
**All cases**	15	20		11	20		26	9		22	11	
**Age**			0.181			0.214			0.179			0.032
<=60	8(34.80%)	15(65.20%)		9(42.90%)	12(57.10%)		17(68.00%)	8(32.00%)		18(78.30%)	5(21.70%)	
>60	7(58.30%)	5(41.70%)		2(20.00%)	8(80.00%)		9(90.00%)	1(10.00%)		4(40.00%)	6(60.00%)	
**Gender**			0.088			0.211			<0.001			0.338
Female	1(14.30%)	6(85.70%)		3(60.00%)	2(40.00%)		1(14.30%)	6(85.70%)		3(50.00%)	3(50.00%)	
Male	14(50.00%)	14(50.00%)		8(30.80%)	18(69.20%)		25(89.30%)	3(10.70%)		19(70.40%)	8(29.60%)	
**Tumor size**			0.843			0.022			0.289			0.026
<5cm	7(41.20%)	10(58.80%)		8(57.10%)	6(42.90%)		12(66.70%)	6(33.30%)		9(50.00%)	9(50.00%)	
>=5cm	8(44.40%)	10(55.60%)		3(17.60%)	14(82.40%)		14(82.40%)	3(17.60%)		13(86.70%)	2(13.30%)	
**Vascular invasion**			0.163			0.660			0.269			0.618
Negative	7(33.30%)	14(66.70%)		8(38.10%)	13(61.90%)		17(81.00%)	4(19.00%)		12(63.20%)	7(36.80%)	
Positive	8(57.10%)	6(42.90%)		3(30.00%)	7(70.00%)		9(64.30%)	5(35.70%)		10(71.40%)	4(28.60%)	
**pTNM**			0.643			0.323			0.402			0.867
1	1(33.30%)	2(66.70%)		0(0.00%)	2(100.00%)		2(66.70%)	1(33.30%)		2(66.70%)	1(33.30%)	
2	4(36.40%)	7(63.60%)		3(27.30%)	8(72.70%)		10(90.90%)	1(9.10%)		6(60.00%)	4(40.00%)	
3	9(45.00%)	11(55.00%)		7(41.20%)	10(58.80%)		13(65.00%)	7(35.00%)		13(68.40%)	6(31.60%)	
4	1(100.00%)	0(0.00%)		1(100.00%)	0(0.00%)		1(100.00%)	0(0.00%)		1(100.00%)	0(0.00%)	
**PDL1-TU**			0.094			0.542			0.248			0.016
0	6(66.70%)	3(33.30%)		3(42.90%)	4(57.10%)		6(60.00%)	4(40.00%)		3(33.30%)	6(66.70%)	
1	9(34.60%)	17(65.40%)		7(30.40%)	16(69.60%)		19(79.20%)	5(20.80%)		18(78.30%)	5(21.70%)	

PD1-IM, Evaluation of PD-1 expression based on staining on immune cells; PDL1-TU, Evaluation of PD-L1 expression based on staining on tumor cells.

### Epstein Barr Virus-Associated Gastric Cancer Patient Outcomes

**Figure 3:**
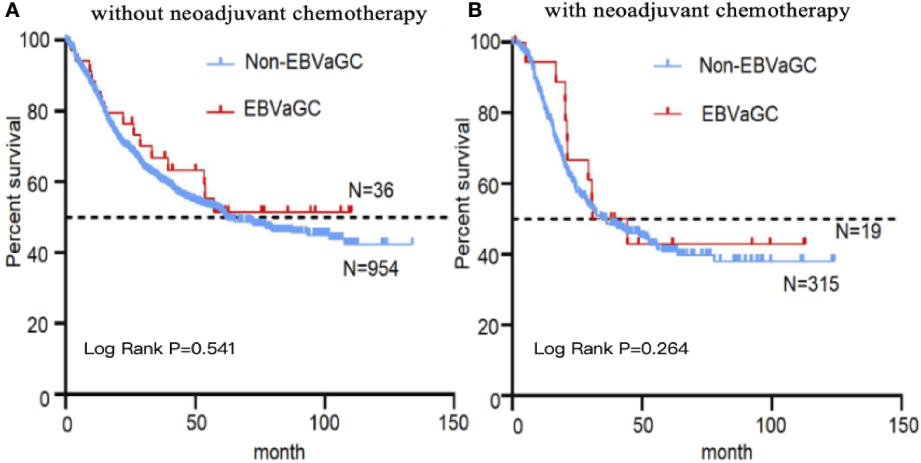
Kaplan–Meier curves stratified by EBV status. Kaplan–Meier survival analysis of EBVaGC and non-EBVaGC patients without **(A)** and with **(B)** neoadjuvant chemotherapy.

The 1,328 patients were subjected to follow-up for 1–133 months, with a median follow-up period of 41 months. Kaplan–Meier survival analysis revealed no significant difference between the EBVaGC and non-EBVaGC patients. However, survival time tended to be longer in EBVaGC patients, especially in those who did not receive neoadjuvant chemotherapy (**Figure 3**).

**Figure 4:**
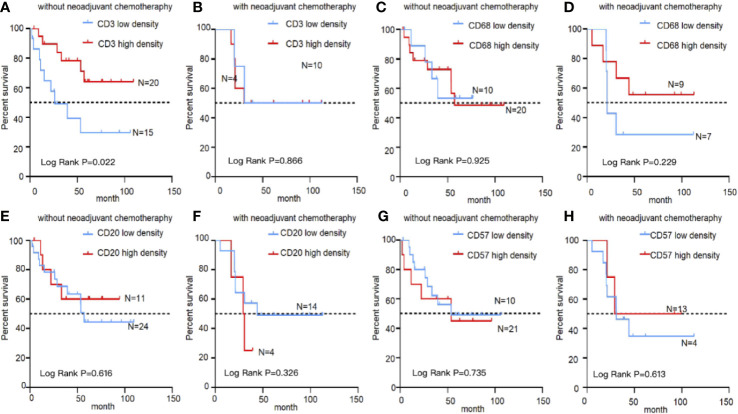
Kaplan–Meier survival curves showing infiltration of immune cells in EBVaGC with or without chemotherapy. Analysis of EBVaGC patients stratified by CD3^+^ T lymphocyte density **(A)** without and with **(B)** neoadjuvant chemotherapy, CD68^+^ macrophages without **(C)** and with **(D)** neoadjuvant chemotherapy, CD20^+^ B lymphocytes without **(E)** and with **(F)** neoadjuvant chemotherapy, and CD57^+^ NK cells without **(G)** and with **(H)** neoadjuvant chemotherapy.

The influence of different immune cells on the prognosis in EBVaGC was investigated. In the treatment-naïve group, survival was better for patients with high CD3^+^ T cell density than that for patients with low CD3^+^T cell density (5-year OS, 0.641 ± 0.120 *vs.* 0.295 ± 0.135, P = 0.022, **Figure 4A**). However, in patients treated using neoadjuvant chemotherapy, the density of CD3^+^ T lymphocytes after chemotherapy did not correlated with the prognosis of EBVaGC (**Figure 4B**). No significant survival differences were evident upon stratification by other immune markers, including CD20, CD57, and CD68 (**Figures 4C–H**).

We conducted univariate and multivariate analyses of the relationship between clinical characteristics and prognosis of patients with EBVaGC. Multivariate analysis using Cox regression models included the patients’ age, tumor differentiation, size, pTNM stage, and CD3^+^ T lymphocyte density. Tumor size and pTNM stage were independent risk factors for the prognosis of EBVaGC patients (**Table 3**).

**Table 3:** Univariable and multivariable survival analysis of EBVaGC cases.

Characteristics	Univariable				Multivariable			
	P-value	HR	95%CI		P-value	HR	95%CI	
			Lower limit	Upper limit			Lower limit	Upper limit
pTNM	0.017	6.157	1.382	27.439	0.005	9.533	1.955	46.482
CD3	0.030	0.314	0.110	0.894	0.010	0.218	0.068	0.699
Tumor size	0.075	2.669	0.906	7.863	0.024	3.952	1.203	12.978

HR, hazard ratio; CI, confidence interval.

## Discussion

This study systematically investigated the relationship between the immune environment and clinical characteristics and prognosis of patients with EBVaGC in a cohort of Chinese GC patients. The pathological types of tumor tissues of EBVaGC patients comprised more poorly differentiated adenocarcinomas. EBVaGC was more common in patients <60 years of age and was associated with less vascular invasions. EBVaGC patients had a better prognosis. In particular, the survival of patients with high CD3^+^ T cells density was significantly longer than those with lower CD3^+^ T cell density. These analyses of the clinical and prognostic characteristics of EBVaGC patients further clarify the role of the immune environment in the development of EBV-related GC. The findings may help in the development of more accurate and efficient treatments.

The proportion of EBVaGC in GC varies between different countries and regions. The rate exceeds 15% in cases in the United States, 2–10% in Asia, and 2–18% in Europe (16). In our study cohort, EBV positive GC patients accounted for 4.1% of the total, which was slightly lower than the proportion of EBVaGC reported in the US and European countries. The low incidence rate necessitated the examination of a considerable number of patients. The cases in the present study were derived from more than 1,328 samples, which is the largest number of cases reported thus far for the overall analysis of the immune microenvironment. A study involving Americans reported the greater prevalence of the EBV subtype in young-onset GC, which might play a key role in the pathogenesis  (17). A Japanese study found that an age <65 years was an independent risk factor for lymph node metastasis in patients with EBVaGC (18). This was consistent with our finding of the negative association of EBVaGC with patient age. A previous study using data from The Cancer Genome Atlas indicated that most of the EBV-positive cases reported in males (81%) and were more often located at the fundus or corpus (62%) (2). A meta-analysis indicated the greater frequencies of EBV infection in GC cases in males and in GCs with lymphoid stroma (19). A study from Taiwan found that 82% of the EBV-associated cases were poorly differentiated. Residual GC (26%) was also significantly associated with EBV infection (20). The present results of the pathological analysis also showed that EBVaGC was related to poorly differentiated cancer. There were no significant differences between EBVaGC and non-EBVaGC in terms of sex and tumor location. A study from Japan found that the probability of lymph node metastasis of EBVaGC was significantly lower than that of non-EBVaGC (4.2 *vs.* 21.9%) (21). We also found that vascular invasion related to metastasis was less common in EBVaGC. Analyses of the function and composition of different immune cells and immune components of EBVaGC are valuable to further the understanding of the unique tumor immune microenvironment of EBVaGC patients. We firstly reported the significance of CD3^+^ T cells density for EBVaGC patient prognosis and suggested that CD3^+^ T cells might play an important role in the immune response of patients with EBVaGC. A distinction should be made between the functions and roles of different types of Th cells, CTLs and Tregs to understand the anti-tumor effect of lymphocytes in EBVaGC. These immune cells are involved in tumorigenesis through different mechanisms. Infiltrating lymphocytes, especially CD8 positive CTLs, appear to exert an anti-tumor effect in EBVaGC (22). Additionally, CD4^+^ CD25^+^ Tregs were significantly increased in EBVaGC compared to EBVnGC (23). The level of tumor-infiltrating lymphocytes and subclassification of EBVaGC significantly influence recurrence-free survival (P = 0.002) and disease-free survival (P = 0.008) in patients with EBVaGC (24). A high density of tumor-infiltrating lymphocytes in GC has been associated with favorable outcomes (25). These results are consistent with our findings. Furthermore, we also found the significantly different prognoses of EBVaGC patients with high CD3^+^ T cells density before and after chemotherapy. The survival time of these patients prior to chemotherapy was reportedly better than that of patients with low density of the cells. However, due to the small number of cases, further verification with more samples is required. The findings suggests that we should make a distinction for patients with high CD3^+^ T cells density in EBVaGC, since these patients may experience limited benefit from chemotherapy. EBVaGC macrophages account for 22% of all intratumoral cells, second only to CD8^+^T cells (28%) (13). In our study, the density of CD68^+^ macrophages in EBVaGC patients was significantly increased. The number of macrophages was related to the size of the tumors. The role of macrophages in EBVaGC may depend on the proportion of M_1_/M_2_ macrophages. It has been reported that EBVaGC has a higher proportion of activating M_1_-macrophages (3) and a higher number of mature dendritic cells than non-EBVaGC (26). A study in Chinese population reported that Lactoferrin reduced synthesis of interleukin-8 and monocyte chemoattractant protein-1 induced by EBV in macrophages via suppression of nuclear factor-kappa B activity (27). Another study reported that decreased proportion of CD204^+^ M_2_ macrophages in EBVaGC was associated with poor prognosis (7). Further analysis of macrophage phenotypic changes in EBVaGC would aid the elucidation of the mechanism of macrophage infiltration, leading to better prognosis.

Some immune cells and matrix components account for only a small portion of the total number of immune cells in the immune microenvironment. However, they are important in the tumor microenvironment by themselves or because they regulate other immune cells. Our results revealed that the number of B lymphocytes and NK cells in EBVaGC was higher compared with non-EBVaGC. Correlation analysis of clinical features more often revealed a high density of CD20^+^ B lymphocytes in female patients. A high density of CD66b-positive tumor-associated neutrophils has been associated with low frequency of lymph node metastasis (28). This suggests that a certain phenotype of neutrophils may be related to the characteristics of tumors that include reduced metastasis and invasion in EBVaGC. The expression of MHC-1 in EBVaGC reportedly increased the level of interferon-gamma in the tumor, which correlated with signatures of increased infiltration by NK cells (29). Our analysis of NK cells in EBVaGC demonstrated that the density of CD57 ^+^ NK cells was related to the patient age, tumor diameter and PD-L1 expression. Several immune molecules are involved in the development of tumorigenesis in EBVaGC. In EBVaGC, mature dendritic cells are in close proximity to tumor cells and appear to have a positive correlation with the abundance of lymphocyte infiltration (30). Higher numbers of dendritic cells were demonstrated in EBVaGC compared to those in non-EBVaGC (9). In epithelial malignancies, cancer-associated fibroblasts are activated fibroblasts in the tumor microenvironment. These fibroblasts are also present in EBVaGC and may play an important role in promoting tumor progression (14).

EBVaGC has a unique tumor microenvironmental characteristic associated with viral infection. This may cause a better response to, and benefit from, chemotherapy in EBVaGC patients, unlike other types of GC. A study from Japan identified genes with higher methylation in EBVaGC cell lines than that in normal gastric cells. Among these methylation-related genes, *ABCG2, AHNAK2, BCL2, FZD1, and TP73* are associated with published evidence of resistance to 5-fluorouracil and cisplatin. Silencing of these genes may be associated with hypersensitivity to chemotherapy (31). In a clinical trial report from Italy, among the one hundred seventy-five cases analyzed, only seven (4%) were EBV-positive and all showed long-lasting and complete responses to first-line chemotherapy with fluorouracil and platinum and a significantly better survival compared with non-EBVaGC patients (32).

Determination of the composition of the EBVaGC tumor immune microenvironment may help to identify people who benefit more from immunotherapy. EBVaGC exhibit high response rates to pembrolizumab that EBVaGC patients may constitute the population that benefits clinically from immunotherapy. In a clinical trial involving sixty-one cases in Japan, all six EBVaGC patients achieved a partial response with a median response duration of 8.5 months (4). However, another research from Japan showed that one in four (25%) EBV^+^ patients treated with nivolumab achieved an objective response. Notably, no EBV patients in our study showed high PD-L1 expression, which might be a factor affecting the benefit of immunotherapy in EBVaGC patients (33). A clinical trial report from Canada based on observations in one patient has supported the role of immunotherapy in EBVaGC and support the rationale for trials of checkpoint blockade in EBVaGC patients in the absence of high mutation burden (3). Ramucirumab, a fully humanized IgG1 monoclonal anti-vascular endothelial growth factor receptor 2 antibody, and checkpoint inhibitors in EBV and microsatellite-unstable (MSI) subtypes have proven beneficial in advanced GC (34). However, whether EBVaGC patients can benefit from immunotherapy remains debatable. Studies on treatment combined with the immune microenvironment are particularly necessary. Identification of therapeutic targets related to immune cells may improve the prognosis of patients, especially in the East Asian population.

Here, we described a large-scale systematic immunological microenvironment analysis of EBVaGC and its relationship with clinical features and prognosis. We analyzed a variety of different immune cells in EBVaGC. T cells and macrophages did not distinguish between cell types with different functional characteristics. For example, the M_1_ and M_2_ polarization of macrophages had opposite effects on tumor growth. Therefore, further differentiation of immune cell subtypes with different functional characteristics will clarify the role of immune cells in the development of EBVaGC tumors. The present study was retrospective and tissue microarrays may not comprehensively represent the original tissues. Prospective studies on immune microenvironment and efficacy in cohort samples of immunotherapy and chemotherapy are needed. The lack of classification data for EBV subtypes and pathological characteristics is another limitation of the present study.

## Data Availability Statement

The raw data supporting the conclusions of this article will be made available by the authors, without undue reservation.

## Ethics Statememt

The studies involving human participants were reviewed and approved by the Beijing cancer hospital institutional review boards. The patients/participants provided their written informed consent to participate in this study.

## Author Contributions

TG, ZML, YF, and ZWL were in charge of the data curation. XJ conducted the formal analysis. MZ, BD, ZWL, YH, and ZYL provided the resources. XJ provided the software. YH, ZYL, XX, SJ, and JJ supervised the study. XJ wrote the original draft. TG wrote, reviewed, and edited the manuscript. All authors contributed to the article and approved the submitted version.

## Funding

The authors are grateful for the financial support extended by the National High Technology Research and Development Program of China (863 Program, No. 2014AA020603) and the Beijing Municipal Science and Technology Project (No. D171100006517000), “Double First Class” disciplinary development Foundation of Peking University (BMU2019LCKXJ011), the Mission Talent Program (SML20151001), Capital funds for health improvement and research (2018-2-103), the Three-year-rotating Budget Program, the Beijing Municipal Commission of Health and Family Planning, the Clinical Medicine Development Special Funding of Beijing Municipal Administration (ZYLX201701), the National Natural Science Foundation of China (No. 81872502, 81972758), the Interdisciplinary Medicine Seed Fund of Peking University (BMU2018MX020), the Beijing Municipal Administration of Hospitals’ Youth Program (QML20181102), the Key Laboratory of Carcinogenesis and Translational Research, the Ministry of Education/Beijing (2019 Open Project-01,02), and the Beijing Municipal Administration of Hospitals Incubating Program (PX2019040), the Science Foundation of Peking University Cancer Hospital (2017-23, 2017-28, 2020-6).

## Conflict of Interest

The authors declare that the research was conducted in the absence of any commercial or financial relationships that could be construed as a potential conflict of interest.
