# Supplementary material for: Clinicopathological and Immunomicroenvironment Characteristics of Epstein–Barr Virus-Associated Gastric Cancer in a Chinese Population
Source: Front Oncol. 2021 Jan 8;10:586752. doi: 10.3389/fonc.2020.586752 (PMC7820894; doi:10.3389/fonc.2020.586752)
Supplement: Supplementary file 1 [file DataSheet_1.docx]

Supplementary Material

# Supplementary Tables

| Supplementary Table S1. Molecular features of GCs according to EBV Status | | | | | | |
| --- | --- | --- | --- | --- | --- | --- |
| Characteristics | N |  | EBV | |  | P-value |
|  |  |  | neg | pos |  |  |
| CD3 |  | |  |  |  | <0.001 |
| Low | 923 | N (%) | 904(97.90%) | 19(2.10%) |  |  |
| High | 306 | N (%) | 276(90.20%) | 30(9.80%) |  |  |
| Missing | 99 | N | 93 | 6 |  |  |
| CD68 |  |  |  |  |  | <0.001 |
| Low | 851 | N (%) | 833(97.90%) | 18(2.10%) |  |  |
| High | 284 | N (%) | 255(89.80%) | 29(10.20%) |  |  |
| Missing | 193 | N | 185 | 8 |  |  |
| CD20 |  |  |  |  |  | 0.883   \|  \| \| --- \| |
| Low | 974 | N (%) | 863(96.30%) | 33(3.70%) |  |  |
| High | 324 | N (%) | 283(96.60%) | 10(3.40%) |  |  |
| Missing | 30 | N | 28 | 2 |  |  |
| CD57 |  |  |  |  |  | 0.779 |
| Low | 950 | N (%) | 915(96.30%) | 35(3.70%) |  |  |
| High | 316 | N (%) | 301(95.30%) | 15(4.70%) |  |  |
| Missing | 62 | N | 57 | 5 |  |  |

| Supplementary Table S2. Correlation between tumor infiltrating immune cell density  and neoadjuvant chemotherapy in patients with EBVaGC | | | |
| --- | --- | --- | --- |
| Characteristics | Neoadjuvant chemotherapy | Without Neoadjuvant chemotherapy | P-value |
| CD3 |  |  | 0.354 |
| Low | 15(42.90%) | 20(57.10%) |  |
| High | 4(28.60%) | 10(71.40%) |  |
| CD68 |  |  | 0.581 |
| Low | 11(35.50%) | 20(64.50%) |  |
| High | 7(43.80%) | 9(56.30%) |  |
| CD20 |  |  | 0.780 |
| Low | 26(74.30%) | 9(25.70%) |  |
| High | 14(77.80%) | 4(22.20%) |  |
| CD57 |  |  | 0.474 |
| Low | 22(66.70%) | 11(33.30%) |  |
| High | 13(76.50%) | 4(23.50%) |  |
|  | Mean±SD | Mean±SD | P-value |
|  |  |  |  |
| CD3 | 23.077±14.353 | 25.75±15.226 | 0.565 |
| CD68 | 9.685±4.718 | 9.852±7.002 | 0.923 |
| CD20 | 5.23±6.989 | 3.35±4.913 | 0.314 |
| CD57 | 4.056±5.57 | 2.525±3.80 | 0.315 |
